# Adapting a community-based ART delivery model to the patients’ needs: a mixed methods research in Tete, Mozambique

**DOI:** 10.1186/1471-2458-14-364

**Published:** 2014-04-15

**Authors:** Freya Rasschaert, Tom Decroo, Daniel Remartinez, Barbara Telfer, Faustino Lessitala, Marc Biot, Baltazar Candrinho, Wim Van Damme

**Affiliations:** 1 Departement of Public Health, Institute of Tropical Medicine, Nationale straat 155, Antwerp 2000, Belgium; 2 Médecins Sans Frontières, Av. Eduardo Mondlane 38, Tete, Mozambique; 3 Médecins Sans Frontières, Rua Damião de Góis 438, Maputo CP 1949, Mozambique; 4 Médecins Sans Frontières, Dupré straat 94 1060, Brussels, Belgium; 5 Ministery of Health, Rua Dos Macondes, Tete, Mozambique

**Keywords:** Community-based care, HIV, ART, Programme implementation, Programme evolution, Patient empowerment, Community participation

## Abstract

**Background:**

To improve retention in antiretroviral therapy (ART), lessons learned from chronic disease care were applied to HIV care, providing more responsibilities to patients in the care of their chronic disease. In Tete - Mozambique, patients stable on ART participate in the ART provision and peer support through Community ART Groups (CAG). This article analyses the evolution of the CAG-model during its implementation process.

**Methods:**

A mixed method approach was used, triangulating qualitative and quantitative findings. The qualitative data were collected through semi-structured focus groups discussions and in-depth interviews. An inductive qualitative content analysis was applied to condense and categorise the data in broader themes. Health outcomes, patients’ and groups’ characteristics were calculated using routine collected data. We applied an ‘input – process – output’ pathway to compare the initial planned activities with the current findings.

**Results:**

Input wise, the counsellors were considered key to form and monitor the groups. In the process, the main modifications found were the progressive adaptations of the daily CAG functioning and the eligibility criteria according to the patients’ needs. Beside the anticipated outputs, i.e. cost and time saving benefits and improved treatment outcomes, the model offered a mutual adherence support and protective environment to the members. The active patient involvement in several health activities in the clinics and the community resulted in a better HIV awareness, decreased stigma, improved health seeking behaviour and better quality of care.

**Conclusions:**

Over the past four years, the modifications in the CAG-model contributed to a patient empowerment and better treatment outcomes. One of the main outstanding questions is how this model will evolve in the future. Close monitoring is essential to ensure quality of care and to maintain the core objective of the CAG-model ‘facilitating access to ART care’ in a cost and time saving manner.

## Background

With the increasing availability of antiretroviral treatment (ART), HIV has become a chronic disease requiring lifelong adherence to treatment. In Mozambique, as in many southern African countries, the health system encounters many challenges to retain patients on ART [1,2]. By the end of 2012, approximately 48% of the people eligible for ART were initiated on treatment [3]. Of them, only 74% were retained on treatment after 12 months [4].

To improve retention on ART, lessons learned from chronic disease care models were applied to HIV/ART care, engaging and giving more responsibility to people living with HIV (PLHIV) in the care of their chronic condition [5,6]. Médecins Sans Frontières (MSF) in collaboration with patients and Ministry of Health (MoH), piloted a community-based model of ART provision and peer support through *Community ART Groups* (CAG). The primary goal was to facilitate access and improve adherence to ART by reducing the patients’ needs to travel every month to the clinic for drug refills [7].

The CAG-model was designed based on the patients’ needs, which are changing over time. Consequently its implementation is a dynamic process, requiring continuous adaptations. This article aims to analyse the evolution of the CAG-model from 2008 to 2012.

## Methods

### Study setting

Tete province counts 2,137,700 inhabitants with an estimated adult HIV prevalence of 7% [8]. Since 2002, MSF has supported the roll-out of the national ART programme. Despite the decentralisation of ART services in 2006, 20% of the ART patients remained lost to follow-up (LFU) [9]. The main barriers were the cost and time investments [10-12].

**Figure 1:**
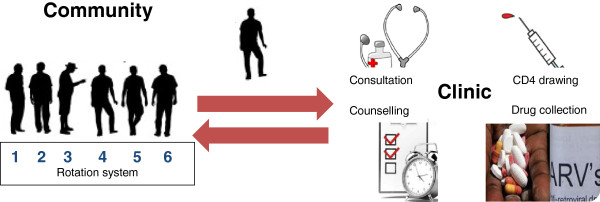
Rotation system of the Community ART groups.

In 2008, to overcome barriers to ART, the CAG-model was implemented involving the patients in the community in standardised care tasks related to their chronic condition. This model was designed to facilitate regular access to lifelong ART and to reduce the workload in the clinics and, by reducing both the need and cost incurred by individual patients to attend clinics every month to collect ART (Figure 1). In 2012, the model was incorporated in the national HIV strategy.

### Study design

A mixed method approach was used, triangulating qualitative and quantitative findings.

### Data collection

A qualitative study using semi-structured interviews was carried out between October 2011 and May 2012 [13,14]. Sixteen focus group discussions (FGD) and 24 in-depth interviews (IDI) were conducted among the five main stakeholders involved in the CAG-model: (a) Patients on ART in groups and in individual care; (b) MoH Nurses; (c) MSF Counsellors; (d) Health authorities; and (e) MSF implementers (Table 1). All FGD and IDI were digitally audio-recorded, transcribed and coded using Nvivo 9 software (QRS International, Doncaster, Vic., Australia).

**Table 1:** Stakeholder groups interviewed in the focus group discussions and in depth interviews

**Stakeholder groups**	**Number of IDI**	**Number of FGD**	**Number of participants**
1. Patients on ART	15	12	79
*In groups*	*4*	*12*	*68*
*Returned to individual care*	*4*		*4*
*Remained in individual care*	*7*		*7*
2. MoH Nurses*	1	2	10
3. MSF Counsellors		2	7
4. Health Authorities (district, provincial and national)	5		6
5. MSF CAG implementer^$^	3		3
**TOTAL**	**24**	**16**	**105**

*MSF appointed counsellors in the main clinics. They have a major role in the daily management of the CAG activities. Whereas in smaller clinics, nurses are responsible for all the CAG-related activities. During the interviews nurses have been divided in two groups: [1] nurses working with counsellors and [2] nurses working without counsellors.

^$^MSF implementers is a core team of MSF, involved in the initial design, implementation and roll-out of the CAG-model.

For the quantitative analysis, routine data was collected on all adult patients registered in a CAG, between February 2008 and December 2012, through individual patient files and group CAG cards, including information on the treatment regimen, drug refills and pill intake. This information was encoded in an electronic database. The clinics were visited periodically to verify the data retrieved.

### Data analysis

Inductive qualitative content analysis was used to analyse the qualitative data. The coded data was condensed and categorised into broader themes [15]. The methodology of the qualitative research is described in more detail elsewhere [16].

**Figure 2:**
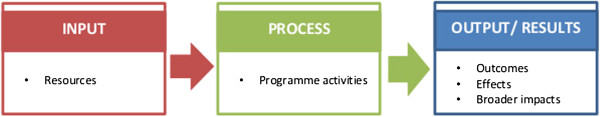
Framework: ‘Input – process – output’ pathway to evaluate adaptations in the CAG-model.

To better understand the evolution of the CAG-model over time, we analysed the data using a framework based on a ‘input – process – output’ pathway, comparing the initial resources, activities and anticipated outcomes with the situation four years after the implementation of the first groups [17-19]. This framework (Figure 2) describes (a) the *input* – the resources made available for the model to function; (b) the *process* – the programme activities; and (c) the *output/results* – the outcomes and effects of the CAG-model.

Variables on individual and group level were collected and categorized. Respectively, the main variables at *individual level* were: [1] gender; [2] age; [3] CD4 count on entering a CAG; [4] ART initiation-date; [5] CAG entry-date; [6] treatment outcomes; and at *group level*: [1] groups with smooth rotation system to collect drugs; [2] groups sharing transport fees; [3] number of members per CAG; [4] type of clinic to which groups are linked. The median and interquartile ranges were presented for numeric variables, and the proportion for categorical variables. Retention in care was defined as patients active on ART in a CAG. Patients transferred out or returned to individual care were considered to be retained up to the date of transfer or return. LFU was defined as being more than two months late for the last appointment or date for drug refill. Data analysis was done using Excel 2010 and STATA 11 (StataCorp LP, College Station, TX, USA).

### Ethics

All study participants gave a written or verbal consent to participate in the study. Ethical approval to conduct the study was obtained from the ‘Ethical review boards’ from the Mozambican MoH and MSF.

### RATS guidelines

The authors confirm that this study adheres to the Relevance Appropriateness Transparency Soundness (RATS) guidelines on qualitative research (http://www.biomedcentral.com/authors/rats) [20].

## Results

**Figure 3:**
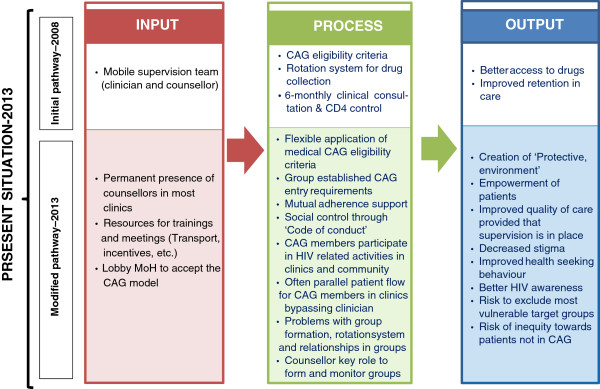
Initial versus modified ‘Input – Process – Output’ pathway, four years after implementation of the CAG-model in Tete, Mozambique.

We analysed the qualitative data retrieved according to the ‘input – process – output’ pathway described above. For each pathway component, we discuss first the initial input, process and anticipated results followed by the additional input, modified process and unintended results reported during the interviews, four year after implementation of the first CAG. Figure 3 compares the two pathways, reflecting on the evolution of the model.

1. **Input**

Initial input

Initially a mobile team, composed of a clinician and counsellor, visited monthly each clinic on fixed days, providing technical support when needed.

Additional input

MSF employed counsellors in most clinics where the CAG-model was implemented. All key-informants considered them as an essential regulatory cadre to link people into groups, and to create and monitor the group dynamic.

Continuous trainings and meetings on the CAG-model functioning and management were said to be organised for CAG members and MoH staff. Moreover, counsellors and health staff highlighted the importance of regular supervision visits to the communities.

All key-informants mentioned the need for additional future resources and support in terms of health staff, drug supply, lab monitoring, tools to monitor the groups, training and transport.

*“There is a need for more counsellors in the clinics because only one cannot manage to respond to all the demands. Because the groups, each time there are more, they are increasing and therefore the human resources especially the counsellors do not manage to respond to the demands.” –* Nurse during FGD with nurses working with counsellors.

2. **Process**

Initial process

*Formation process* - The mobile team appointments were organised as ‘group sessions’ during which newly diagnosed HIV-positive patients were introduced to patients already on ART. Patients stable on ART were invited to form small groups according to their geographical residence. Interested candidates were screened to ensure they were more than six months on ART, had a CD4 count >200 cells/mm^3^, and no active opportunistic infections. Initially groups up to 20 patients were formed; in order to improve the functioning, the groups were limited to maximum six people.

*Functioning of the groups* - A rotation system was installed whereby members took turns visiting the clinic monthly, having a medical consultation and a CD4 sample taken, and collecting the drugs for their fellow group members. Individual and group cards were used to check the pill counts and the adherence of each member. When a member was ill, (s)he was expected to consult the clinic irrespective of the scheduled appointments.

*“Today is my turn to collect drugs but my colleague is ill, though his turn to collect drugs already passed. I have to give him the ART cards of the group so he can go again to the hospital to use the opportunity to receive a medical consultation that day…” –* CAG member during FGD with CAG members from rural areas.

Modified process

*Formation process* - All key-informants agreed that most groups were formed in the clinics with the assistance of a counsellor or nurse, who presented the potential members to each other. Although some members mentioned approaching others in the community to form groups or bringing a list of candidates to the clinics. Sometimes patients joined groups up to ten members before splitting into two groups. Likewise, more experienced patients on ART were asked to form groups with newcomers.

*“…when we identify six people, we go to the clinic to talk to the counsellor and tell him we want to form a group. This one and this one… The counsellor will write all the names down to form a group” –* CAG member during FGD with members from semi-urban areas.

Once formed, each group elected a group leader, who acted as the group’s reference person, responsible for the organisation and the information exchange between members. According to the CAG members, the group leader was often considered as a father or mother figure, or a protector of the group.

“*The group leader needs to be someone who always complies with the recommendations the doctors give. It also needs to be someone who helps and counsels others, encourages colleagues to take their drugs correctly. It has to be a person who is compassionate. When problems occur, (s)he has to be capable to say ‘do this, this or this’. The person has to be an example for the group…”* – Group leader during FGD with group leaders of remote areas.

Health staff and CAG members reported that several groups were composed of only two or three members, often the family nuclei. The quantitative analysis confirmed that 44% of the groups counted less than four members.

According to the CAG members the most frequent problems that occurred when forming groups were: people joining without the knowledge of the other members or without understanding the CAG functioning and responsibilities. Some reported people being refused access to drugs when not being in a CAG, though all key-informants agreed that patients should never be forced to join.

“*In my neighbourhood, it happened that some people refused to join groups. But when they arrived at the hospital to collect drugs they were sent back […] and told they first had to join a group to be able to receive drugs*…” *–* CAG member during FGD with CAG members of semi-urban areas.

CAG members, health staff and MSF implementers confirmed that the established medical eligibility criteria were not always respected. They had some discrepant opinions concerning the required duration on ART, ranging from three to eight months, and the need to be clinical stable before joining a CAG. Some nurses and health authorities strongly defended the need to be adherent prior to joining a CAG, while others opted to target patients at risk for poor adherence such as TB patients, pregnant women and/or children. Quantitative analysis showed a median follow-up time on ART of 19 months (IQR,10-29), a median CD4 count of 385/mm^3^ (IQR,258-560) and 17% of the patients having less than six months on ART before joining a CAG.

CAG members and health staff reported that several groups imposed additional CAG entry requirements e.g. to be physical well and able to participate in the rotation system. Although belonging to the same social class was not a necessity, some people from higher socio-economic class preferred to form separated groups with people from the same rank.

*“We have to see if a person can commit with his/her body and soul, if (s)he can form a group, if (s)he can help the other members of the group […], so when we see that this person does not have the requirements, (s)he cannot enter, because (s)he is not able to help others…” –* Group leader during FGD with group leaders from semi-urban areas.

*Functioning of the groups* - Most key-informants reported that the group members attended regular meetings in the community, during which they performed pill counts, offered adherence support, discussed problems and shared experiences. Also patients not eligible for a CAG could participate in these meetings. This group dynamic seemed quite similar in all groups, independent of the context.

“*We in our group, we do not have major problems because we meet regularly to share ideas and discuss, we visit each other at home, we counsel each other on how to deal with our situation…*” – CAG member during FGD with CAG members from rural areas.

CAG members, health staff and MSF implementers mentioned that to control the behaviour of fellow CAG members, most groups installed a ‘code of conduct’, often compared to Nyau, a cultural ‘secret society’ [21]. The two main rules highlighted were (a) keeping secrecy in a CAG and (b) not allowing members to consume alcohol, because they feared it might hamper the secrecy rules; nor smoke or eat certain species (e.g. chili pepper), as these were thought to jeopardise their health. Some patients in individual care reported examples of secrecy rules not being respected. Members not obeying to the rules were counselled and could be asked to leave the CAG. MSF implementers however stressed that these rules need to be closely supervised to avoid misuse or too rigid application.

*“Because in group, all secrets only belong to the group. You cannot spread a secret of the group outside, no!”* – IDI with CAG member from rural area.

In some clinics with a counsellor, CAG members reported a parallel patient-circuit. Often they were directly seen by the counsellor, who verified the ART cards and weights, and distributed the drugs. Most of the counsellors were convinced clinical consultations were not required on a regular base.

*“Nowadays what changed in the hospital is that when we go to the hospital, we do not have to wait in the queue, when we arrive, arrive there… we only have to hand in the ART cards, they do a pill count… afterwards you receive the drugs, put them in your bag and you are free to go without any delays. We receive the drugs and take them to the group…” –* CAG member during FGD with CAG members from semi-urban areas

All key-informants confirmed that through the CAG-model patients obtained a more active role in different health activities i.e. giving health talks, packing drugs, performing pill counts, counselling patients, organising patient files, sensitizing people in the community to attend general vaccination campaign, tracing defaulter and some even mentioned being involved in HIV testing.

*Problems encountered* - CAG members, health staff and MSF implementers highlighted the infrequent participation of members in the rotation system as one of the main problem. Quantitative data confirmed that in 43% of CAGs, not all members participated regularly in the rotation system. The main reasons mentioned were lack of time or money, being sick, laziness, shame, wanting to hide or illiteracy.

*“Where lots of people gather problems always occur… we have some members who are not disciplined, they prefer that the others go and collect the drugs for them, they do not want to participate…” –* Group leader during FGD with group leaders from rural areas.

Also the lack of privacy in the clinics was felt as an ongoing problem by most CAG members, because often the consultations of CAG members took longer, exposing them to gossip. On the contrary, others thought the CAG-model offered more privacy. Other frequently mentioned problems were relationship problems between group members: marital problems between couples in groups, members not wanting to disclose or not being adherent, a lack of confidence in each other, not contributing money and not obeying to the group rules, etc.

3. **Output/results**

Anticipated results

*Practical benefits* - The major benefit of the CAG-model remained the time and cost savings, allowing people to attend other social activities. Quantitative data revealed that in 28% of the CAG members shared transport costs.

“*For myself, … what changed for me is: […], before we did not have time for other activities, we also had lots of cost to pay transport, so now that we entered in groups, I can see that lots of things changed for us. Money for transport costs is decreasing, with the rotation system of six people, five months pass during which you have time for other activities, only the sixth month you have to go to the hospital…”* – Group leader during FGD with group leaders from rural areas.

*Treatment outcomes* - The better access to drug refills contributed to improved retention on ART. Many CAG members thought that through the CAG-model the mortality decreased remarkably in the communities. The quantitative analysis showed that by December 2012, 6,159 patients on ART joined a group, of whom 431 (6,9%) were transferred out, 15 (0,2%) were LFU and 242 (3,9%) died, with an overall retention rate of 95.7% after a median follow-up time of 19 months in CAG.

*We can see that this (CAG) is very good because many people are no longer lost to follow up or no longer die because people are united, they contribute and manage to go to the hospital.”* – IDI with CAG member from semi-urban area.

### **Unintended results**

*Psychosocial benefits -* All key-informants agreed that being in a CAG, patients understood better the importance of lifelong adherence to treatment and had more confidence in their regular drug supply.

“*The advantage of being in group is the rotation system to collect drugs. The day I cannot go to the hospital, I have the certainty my colleague will bring me the drugs to my home. Therefore I think there are more advantages of being in groups than in individual care…*” – Group leader during FGD with group leaders of semi-urban areas.

CAG members, health staff and MSF implementers felt that the CAG-model helped breaking the patients’ isolation, knowing they were many in the same situation. The model was thought to create a strong bond between members. They often referred to it as a new family or church. CAG members mentioned they felt more respected, mentally stronger and were less sensitive to gossip. They often helped each other to disclose their HIV status to their families.

*“Also through the groups, we gain friendship, we became partners, and we remain aware that in reality…, this disease is an important disease, for which we have to watch out for, it is not whatever disease. We also gain awareness that in reality it is not only me, I should not feel shame. I can summarise that being in groups made an end to all these problems…” –* Group leader during FGD with group leaders from semi-urban areas.

*Benefits at health facility level* - All key-informants perceived an overall improvement in the quality of care since the implementation of the CAG-model. Nurses working with counsellors and district health authorities mentioned a significant reduction of the workload, allowing them more time to attend ill patients. Nurses working without counsellors however reported no notable change as the administrative work increased. Health staff found that through a direct information loop, patients were better monitored. Both CAG members and health staff stated that their relationship was strengthened, considering themselves as colleagues, family or friends.

“*For example, when we have a patient lost to follow up from a particular area where we have two or three CAGs… when these groups or their representatives come to the clinics, we discuss these issues… the representative brings the necessary information related to this patient lost to follow up.”* – IDI with District health authority.

*Broader impact on the community* - Most key-informants reported a significant reduction of HIV stigma in the communities. Patients became more vocal and confident to discuss and negotiate their own and peers’ health. Through the patients’ active role people had better access to health information in the community. Subsequently the HIV knowledge and awareness, and health seeking behaviour - including increased uptake of HIV testing services improved. Nevertheless, some CAG members highlighted problems of ongoing stigma mainly when trying to sensitize people for HIV testing.

*"What has changed, is before when people were not in groups, people did not trust us, they mistrusted us because we were not together, we were separated and isolated, without knowing that we were in the same situation. In the community, there were people who used to talk about us all the time. Now that we are together, […] they know that what we have can affect everyone, so now the gossip seems to have stopped, because it involves a lot of people." –* Group leader during FGD with group leaders from rural.

*“We saw that during the last years the patients before going to the health facilities went to the traditional healers, but today we can see little by little a change in people going first to the hospital…” –* IDI with MSF implementer.

## Discussion

This data analysis highlights the main modifications in the ‘input – process – output’ pathway between the implementation of the first CAG and its functioning four years later.

Input-wise, to ensure optimal functioning of the CAG-model a ‘regulatory cadre’ was needed to link patients in groups, to monitor and to supervise the group functioning. In addition, a good monitoring and information system, and regular trainings were considered key.

During the process, a number of adaptations seemed essential to respond to the patients’ needs and to reinforce the functioning of the CAG-model. Medical eligibility criteria were used more flexible patients were gradually more engaged in their healthcare, ranging from standardised HIV-related tasks (ART refill, adherence support, outcomes reporting) to an active collaboration in the healthcare management in the clinics and community. Nevertheless, the irregular participation in the rotation system remained unresolved. Furthermore, the imposed entry requirements and code of conduct need close monitoring as they might jeopardise the quality of care and the accessibility of the CAGs.

Through the patients’ central role in the CAG-model, the output exceeded the initial expectations. Besides being a strategy to easily access the monthly drug refills, using cost and time saving strategies, the CAG-model created a social dynamic inside and beyond the groups. Patients not only felt stronger but also obtained more respect in the communities, reinforcing the patients’ confidence and ability to cope with their condition. Likewise, the CAG-model offered a protective environment where patients shared and discussed their problems and concerns.

Last but not least, the CAG-model resulted in improved treatment outcomes. A recent analysis comparing the outcomes of patients in a CAG and in individual care, in 10 Mozambican provinces, showed similar outcomes: a LFU rate at 12 months of 11% and 26% respectively; and mortality rate of 1% in both cohorts. Overall attrition at 12 months was 12% in the CAG cohort compared with 28% in the non-CAG cohort [22].

To ensure lifelong adherence to treatment, the care needs to be incorporated in and adjusted to the patients’ daily life [5]. The more patients are given the possibility to make an informed choice in the care of their chronic condition, the more determined they will be to change their behaviour required to adhere to treatment [23-25].

Nevertheless, only few studies on active PLHIV involvement in ART delivery are found in the literature [26]. PLHIV are most commonly involved in tasks related to education, counselling, adherence support and patient tracing. Few examples are available of involving patients in ART distribution, planning, coordination and monitoring of activities [27]. In Uganda and Kenya, peers and volunteers were responsible for a fixed number of patients, which they visited regularly at home to distribute ART and offer adherence support. In South Africa, patients were organised in community clubs. Two to four monthly, they met in the clubs to collect their drugs and discuss ongoing problems. All pilot projects provided promising results. In Uganda, similar attrition rates were reported among patients in the community- and clinic-based model [28]. Over 80% of patients followed in community-based care experienced more family and community emotional support [29]. In Kenya, at 12 months, 5% patients LFU were reported in both patients groups and 1% and 0% deaths in the community- and clinic-based group respectively [30]. In South Africa, patients followed up in clubs were found less at risk to be LFU (RR:0.25,95%CI:0.14–0.41) and to have a virological rebound (RR:0.35,95%CI:0.31–0.40) [31].

To allow increased patient responsibilities and involvement in decision-making process, some basic buildings blocks are required: (a) a good information flow, (b) a trust relationship, (c) a power shift between health staff and patients, and (d) problem-solving and decision-making skills [23,27,32]. These skills together with a sense of ownership are crucial for the patients to recognise and deal with their conditions, and not to perceive decisions and solutions as imposed obligations. Also the feeling of being trusted and respected is important as people will be more likely to adapt their behaviour when promoted and supported by confidants. Therefore peer support, based on sharing their day-to-day experiences, becomes a cornerstone in HIV care [33-35]. In addition, community engagement can help to disseminate information in the community, reach people otherwise not reached [36]. These modifications require a good organisation and competent staff. Health staff plays a crucial role to train and coach patients in these new skills [37]. Table 2 highlights the responsibility shifts between the individual and community-based models.

**Table 2:** Shift patients’ responsibilities between individual care and CAG ART delivery models

**Care aspects**	**Individual ART delivery**	**CAG-model**
**Information flow**	One way communication	Open communication between HCW and patients
**Motivation**	External, having to comply to instructions	Internal, patients gain better understanding of treatment and importance of adherence
**Training and education**	Disease oriented knowledge transfer	Transfer of problem-solving skills to cope with their chronic condition through sharing of day-to-day experiences
**Relation between HCW and patients**	HCW considered as superior, difficult to approach	Trust relationship, HCW and patients considered each other as friends/partners
**Relation between peers**	Patients feel isolated	Strong mutual peer support based on day-to-day experiences
**Power shift**	Patients follow passively instructions of HCW	Patients are actively involved in their health decision making
**Solutions**	Offered by HCW	Patients search for solutions themselves with the support of peers and HCW
**Needs**	Identified by HCW	Identified by patients
**Responsible for treatment outcomes**	HCW	Patients and HCW share responsibilities
**Information dissemination**	Limited to the consultation	Reaching the broader community

Moreover, implementation of innovative models is a dynamic process requiring regular adaptations and adjustments to continue to meet the beneficiaries’ needs and create a sense of ownership [38,39]. The implementation process of the CAG-model illustrates how a proposed template was implemented and evolved over time in diverging ways according to the patients’ needs. The more flexibility for modifications is allowed, the more likely a model will be adopted by the beneficiaries and community. Some core elements however should be safeguarded, not to weaken the initial objectives of the model. In the future, it is likely that the CAG-model could evolve to an open model where certain patients can easily alternate ART access between individual care and a CAG. Such an evolution would create an even greater need for a ‘regulatory function’ and good information system to facilitate these switches. Further mixed methods research will be required to define the extent of simplified ART delivery, transferring key responsibilities for patients along the care cascade versus maintaining a standard quality of care.

The strengths of the study are the vast number of stakeholders interviewed and the accuracy of verification process during data collection, transcription and translation to assure quality of the data and. The major limitations are first, the possible recall bias of participants interviewed. Second, a selection bias needs to be taken into account as patients in a CAG might be clinically more stable on ART. Third, as priority was given to the language skills of the local research team compared to their prior experience in qualitative research, we sometimes had to compromise on the iterative reflection process.

## Conclusions

The findings highlight the need for a flexible approach of community-based ART delivery models adapted to the local context, resources and needs. One of the main outstanding questions is how this group dynamic will evolve in the long term. Further future modifications will likely be required to adapt to the changing needs and context, to motivate patients and to avoid participation fatigue [40]. Likewise, close monitoring is essential to maintain the core objective of the CAG-model ‘facilitating access to ART care’ in a cost and time saving manner. Moreover, a ‘regulatory cadre’ remains needed to form and monitor groups, and to ensure quality of care and equal access to groups.

## Competing interests

The authors declare that they have no conflicts of interest.

## Authors’ contributions

FR conceptualized the study and wrote a first draft, which was edited by all authors. BT and FL collected the data, and coded and analysed the data together with FR. TD, WVD, DR, MB, BT and FL checked scientific soundness and reviewed the manuscript several times. All authors (WVD, FR, TD, DR, MB, BT, FL and BC) contributed to the intellectual content of this article. FR and TD finalized the manuscript. All authors read and approved the final version prior to publication.

## Pre-publication history

The pre-publication history for this paper can be accessed here:

http://www.biomedcentral.com/1471-2458/14/364/prepub
